# Supplementary material for: Transportan Peptide Stimulates the Nanomaterial Internalization into Mammalian Cells in the Bystander Manner through Macropinocytosis
Source: Pharmaceutics. 2021 Apr 14;13(4):552. doi: 10.3390/pharmaceutics13040552 (PMC8070997; doi:10.3390/pharmaceutics13040552)
Supplement: Supplementary file 1 [file pharmaceutics-13-00552-s001.zip › SUPPLEMENTARY MATERIAL/pharmaceutics-1175264 - supplementary for proof.docx]

Supplementary Materials: Transportan peptide stimulates the nanomaterial internalization into mammalian cells in the bystander manner through macropinocytosis

Yue-Xuan Li, Yushuang Wei, Rui Zhong, Ling Li and Hong-Bo Pang

| **Publisher’s Note:** MDPI stays neutral with regard to jurisdictional claims in published maps and institutional affiliations.  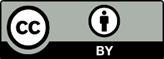  **Copyright:** © 2020 by the authors. Submitted for possible open access publication under the terms and conditions of the Creative Commons Attribution (CC BY) license (http://creativecommons.org/licenses/by/4.0/). |
| --- |


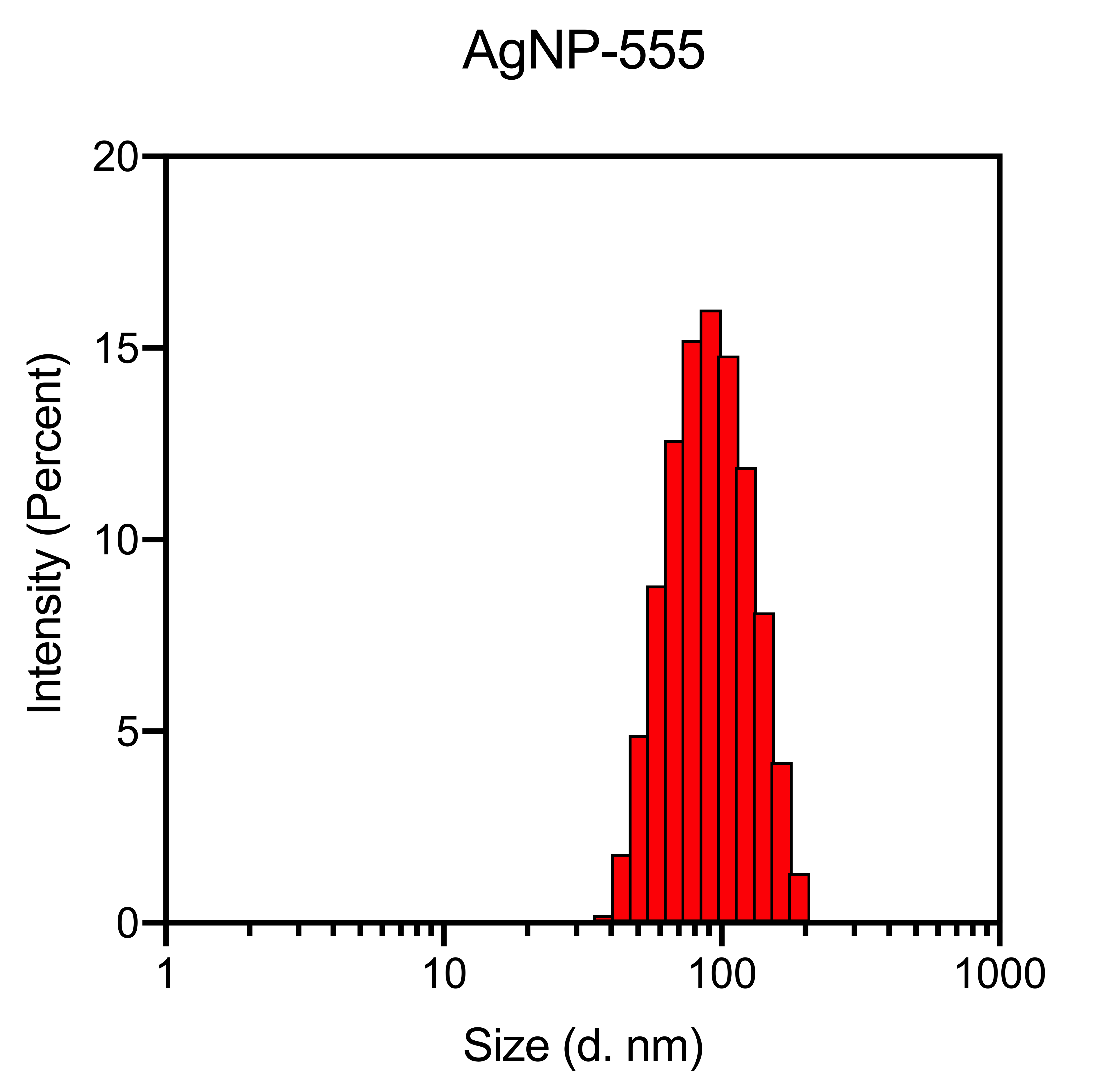

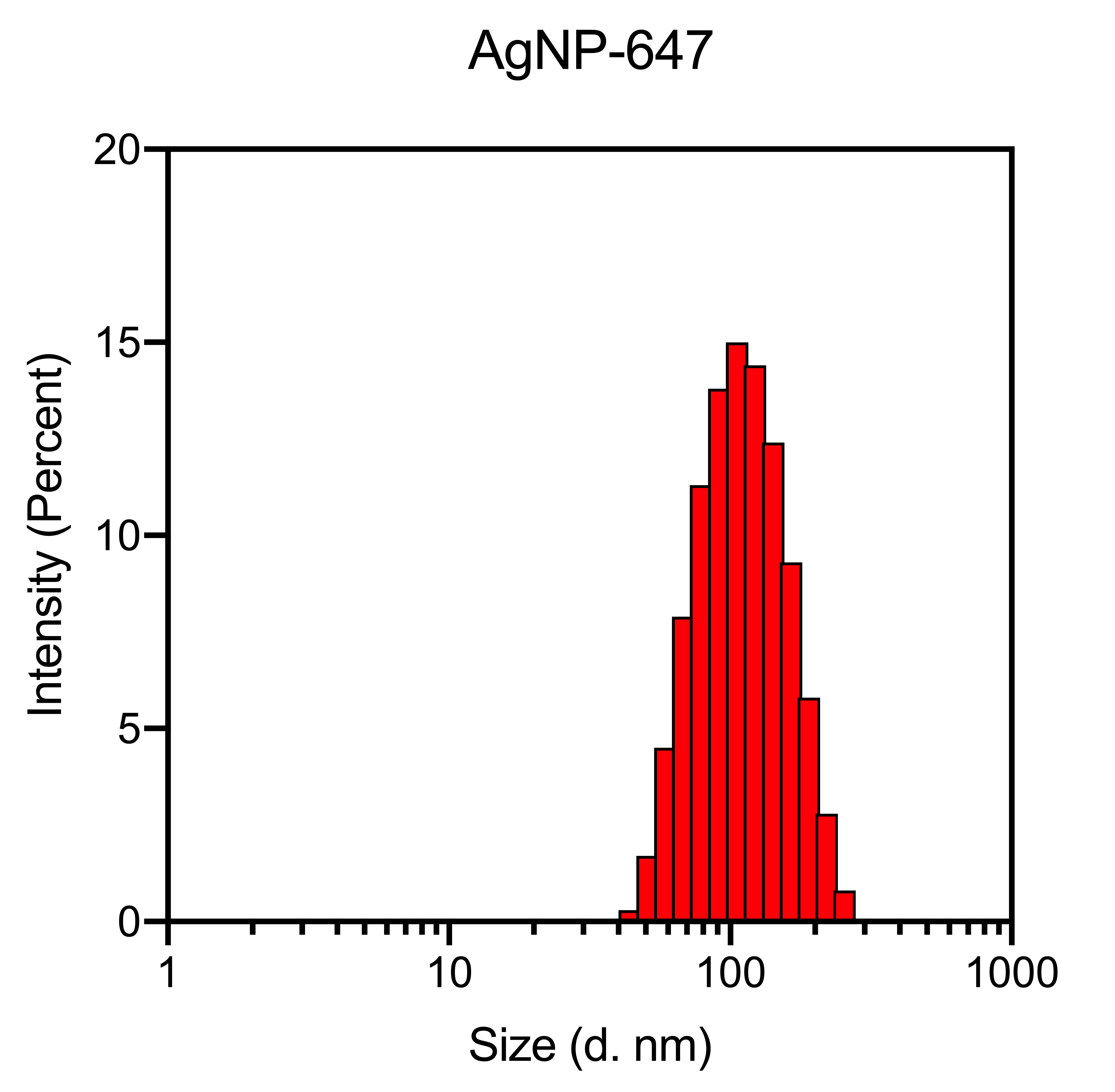

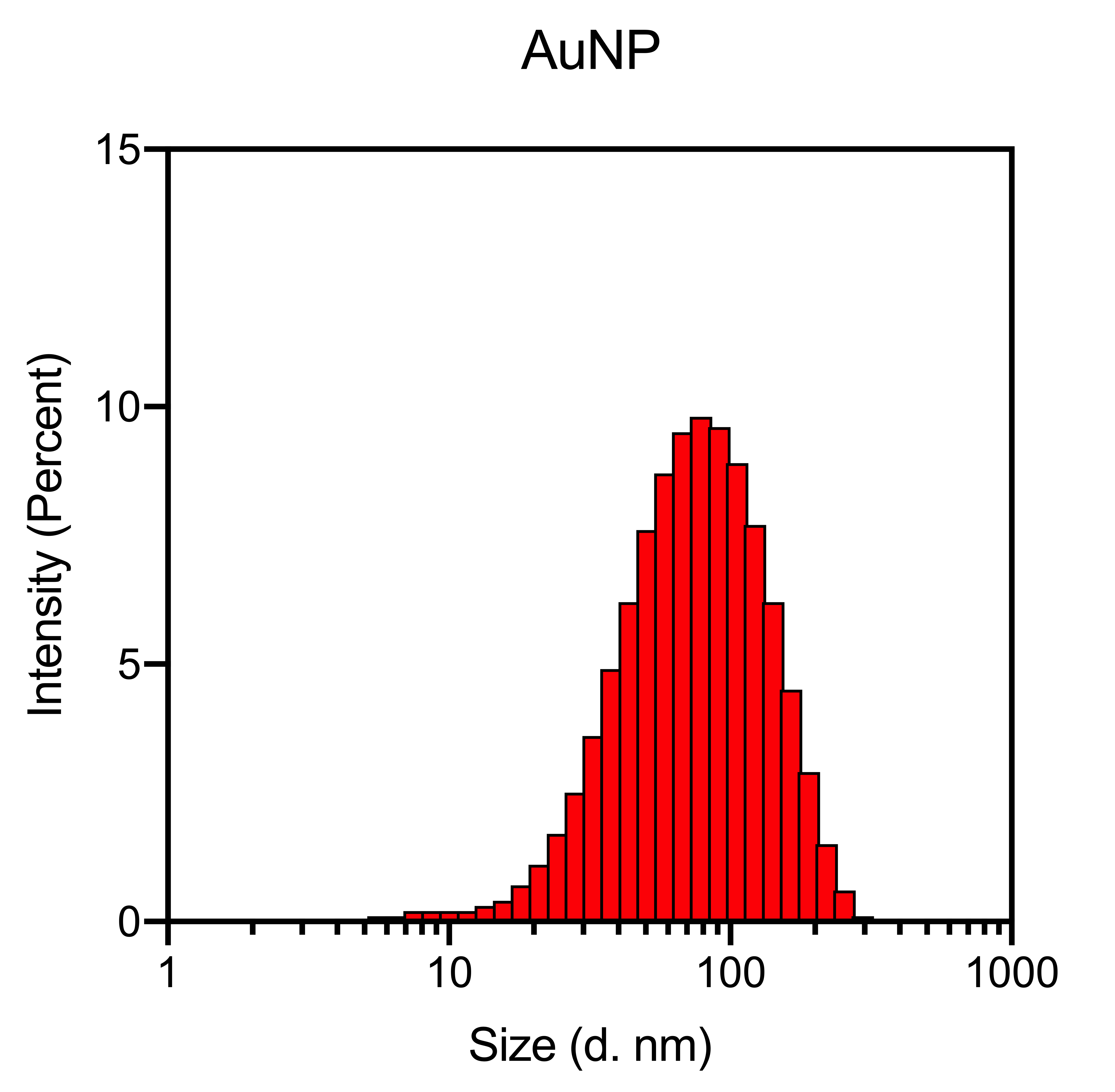

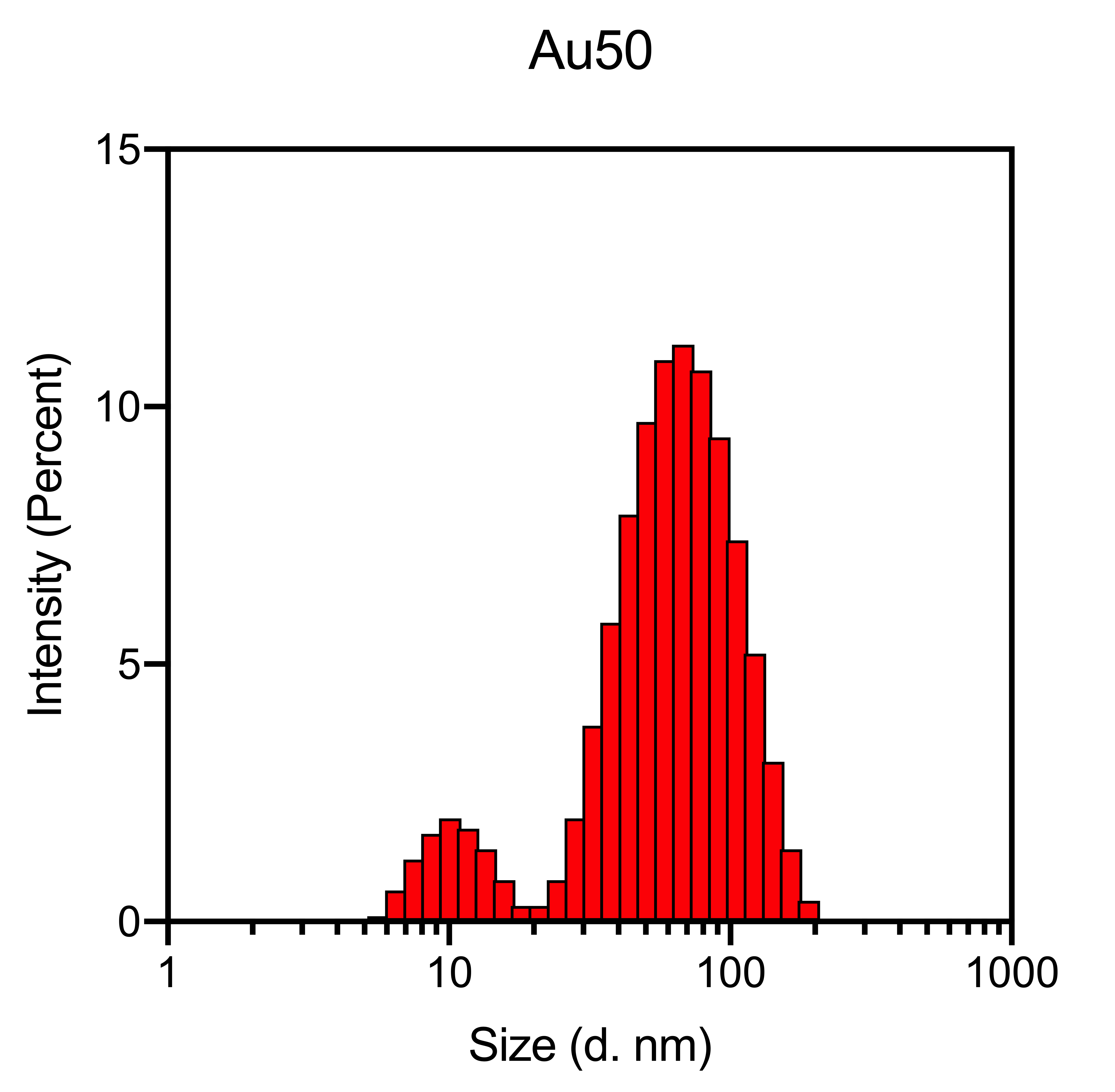

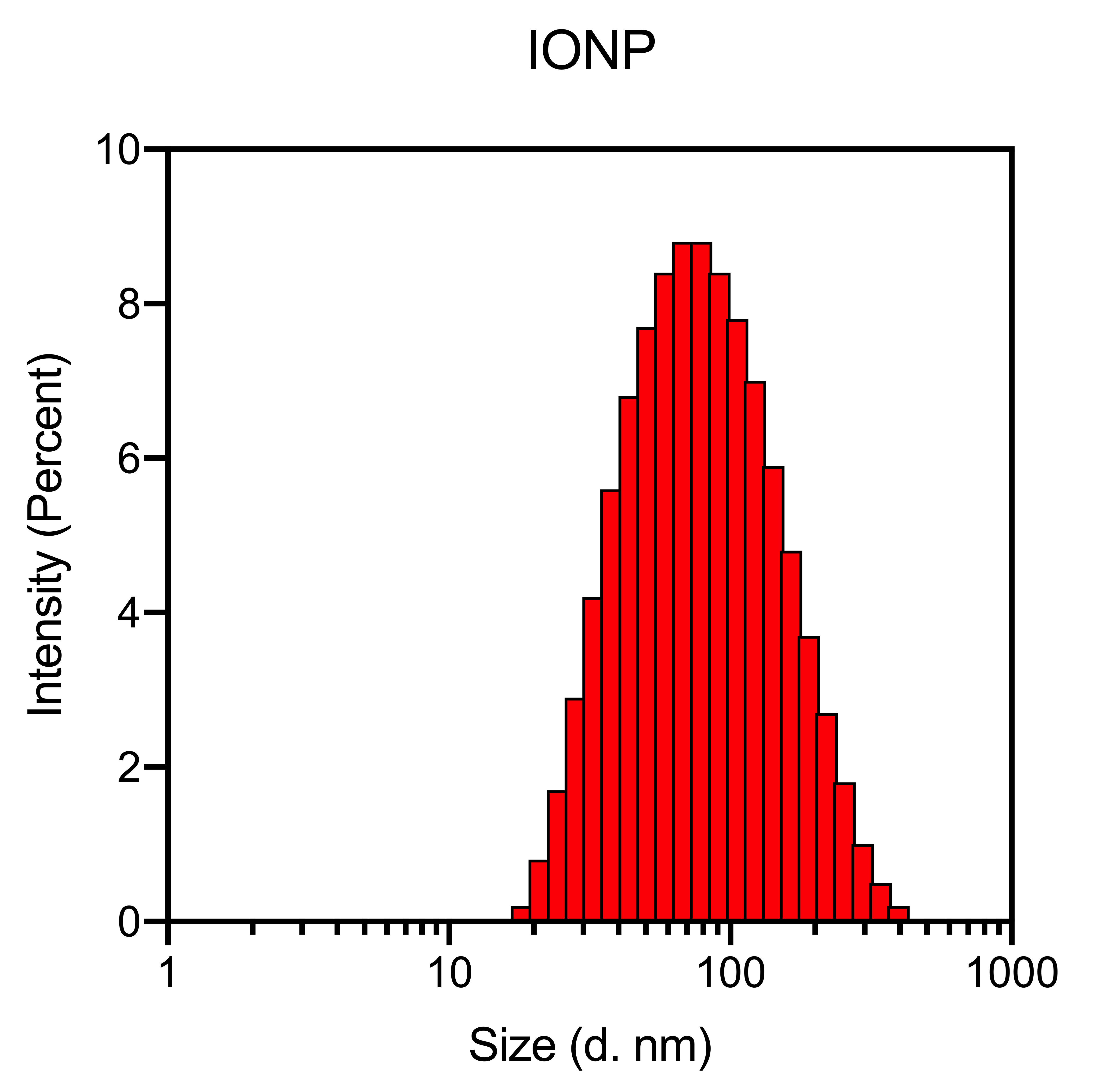

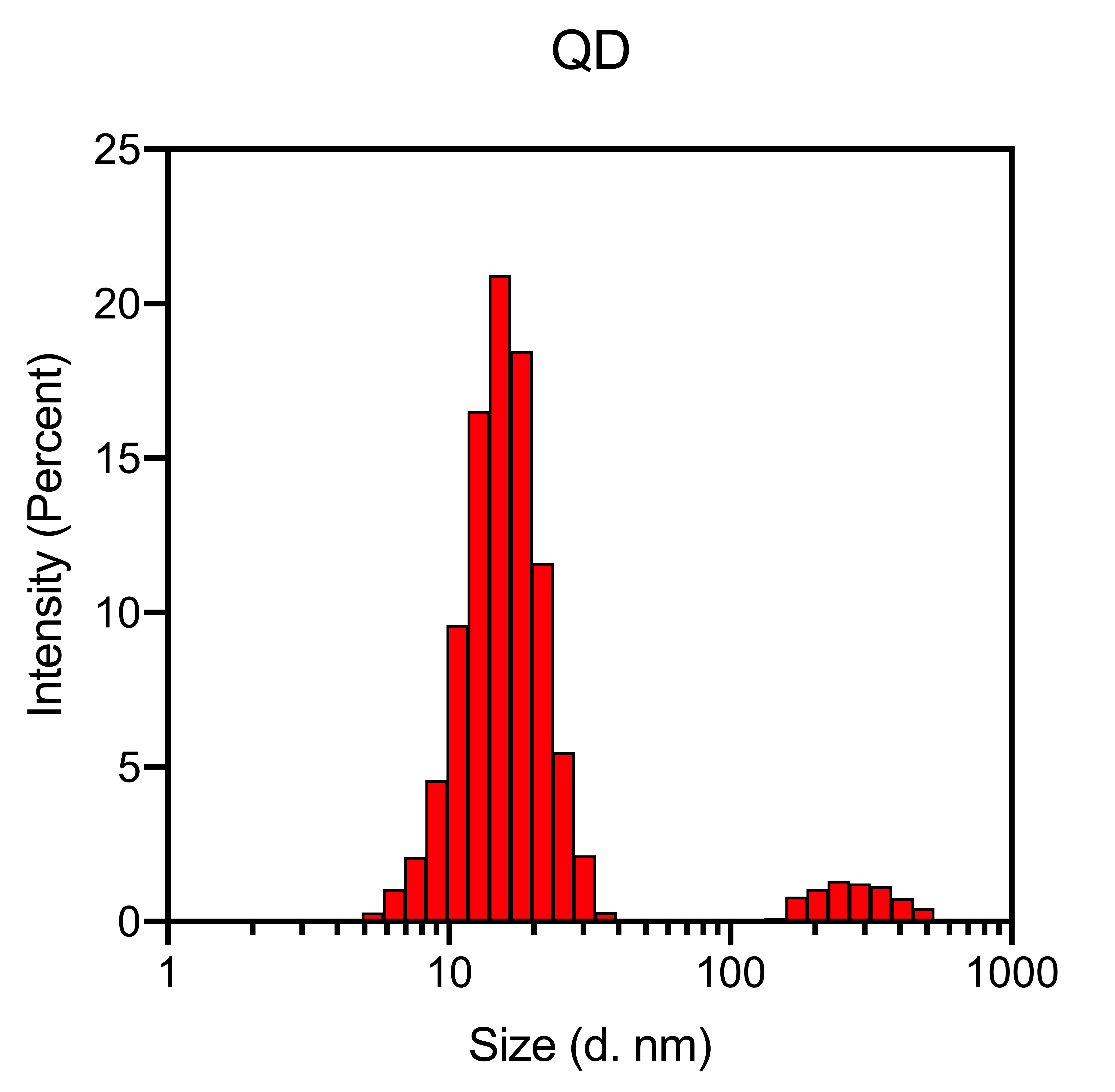


**Figure S1.** Particle size distribution profiles. Silver, gold, iron oxide nanoparticles and quantum dots were synthesized as described in the Methods. Their size distribution by intensity was analyzed with a Nano ZS particle analyzer (Malvern, UK). See Table 2 for their z-average size and PDI. *AgNP* silver nanoparticle, *AuNP* gold nanoparticle, *IONP* iron oxide nanoparticle, *QD* quantum dot.


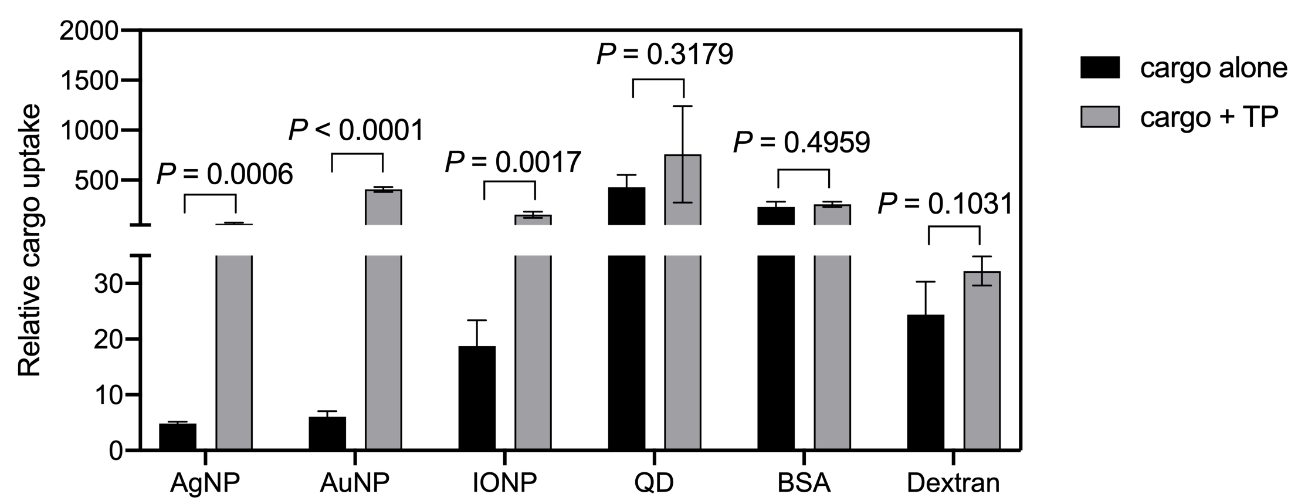


**Figure S2.** TP-induced bystander uptake in HUVECs. Bystander cargo (AgNP, AuNP, IONP, QD, BSA and dextran) was either mixed with human umbilical vein endothelial cells (HUVECs) alone or together with TP peptide at 37 °C for 1 h, as described in the Methods. After etching or washing, the fluorescence intensity of internalized cargo was quantified by flow cytometry and normalized to that of corresponding cells alone (y-axis). Error bars, mean ± standard deviation (s.d.) (*n* = 3). Two tailed Student’s t-test was performed. P value was indicated and considered significant when *P* < 0.05.

**A**


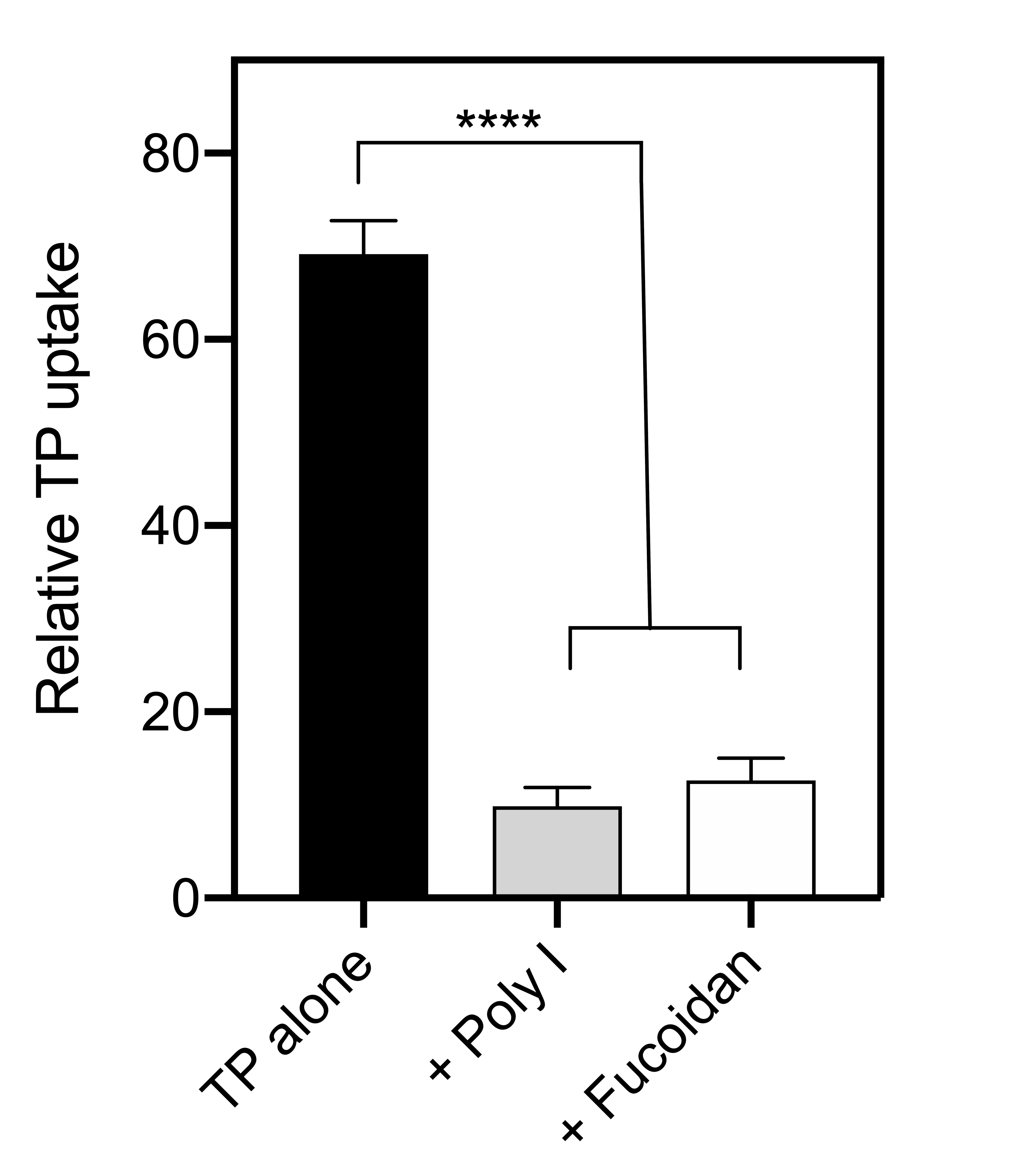


**B**


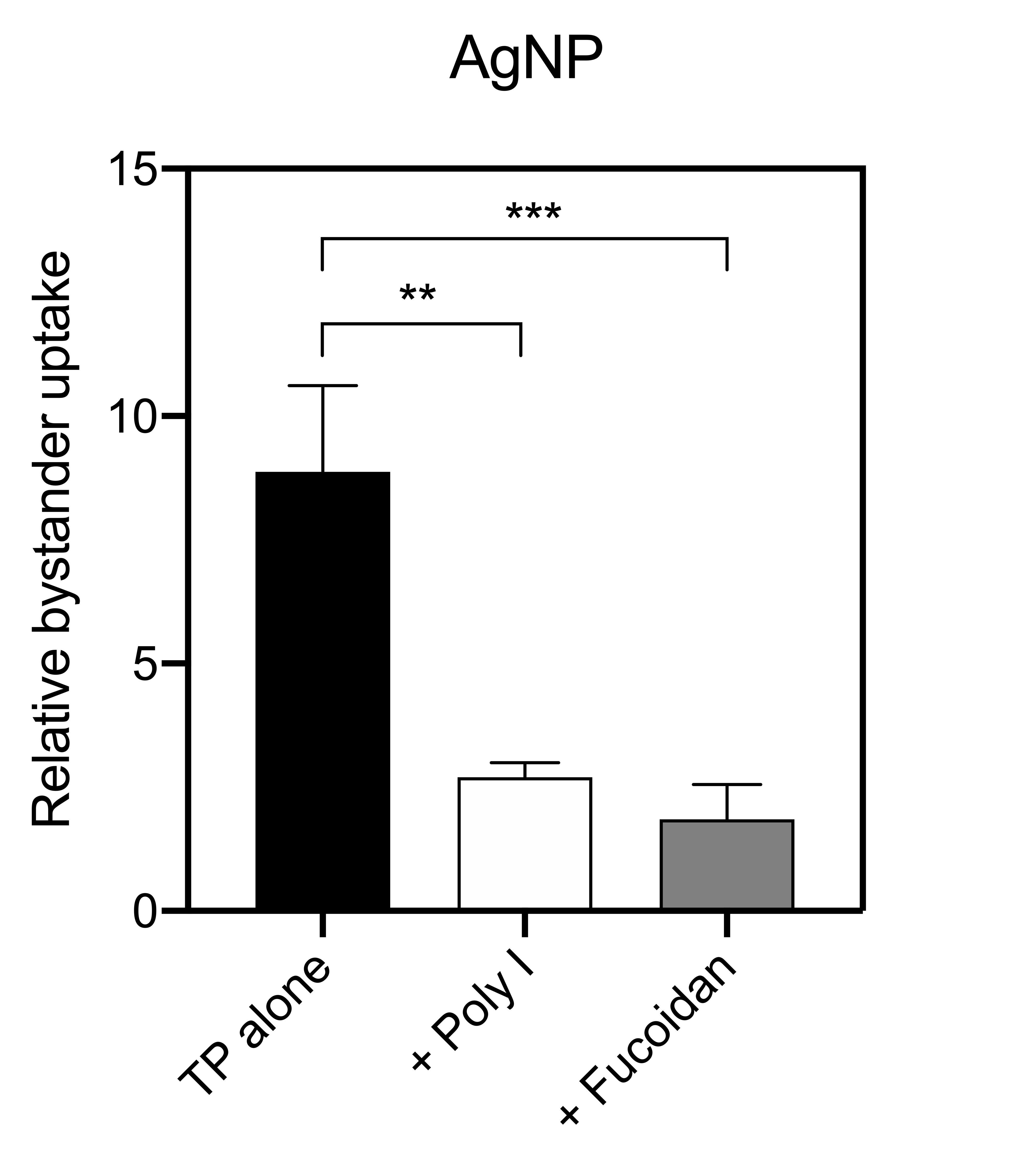

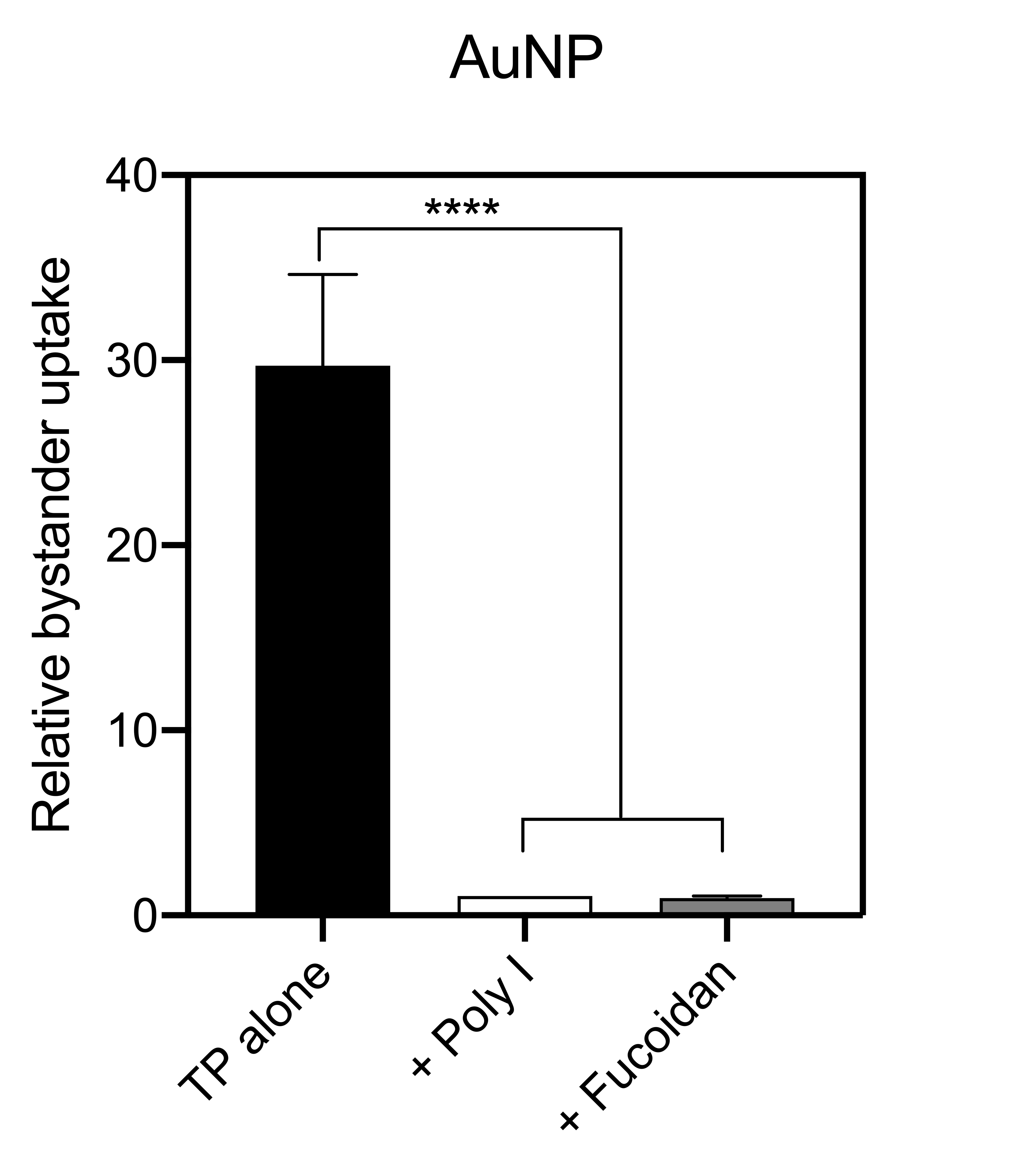

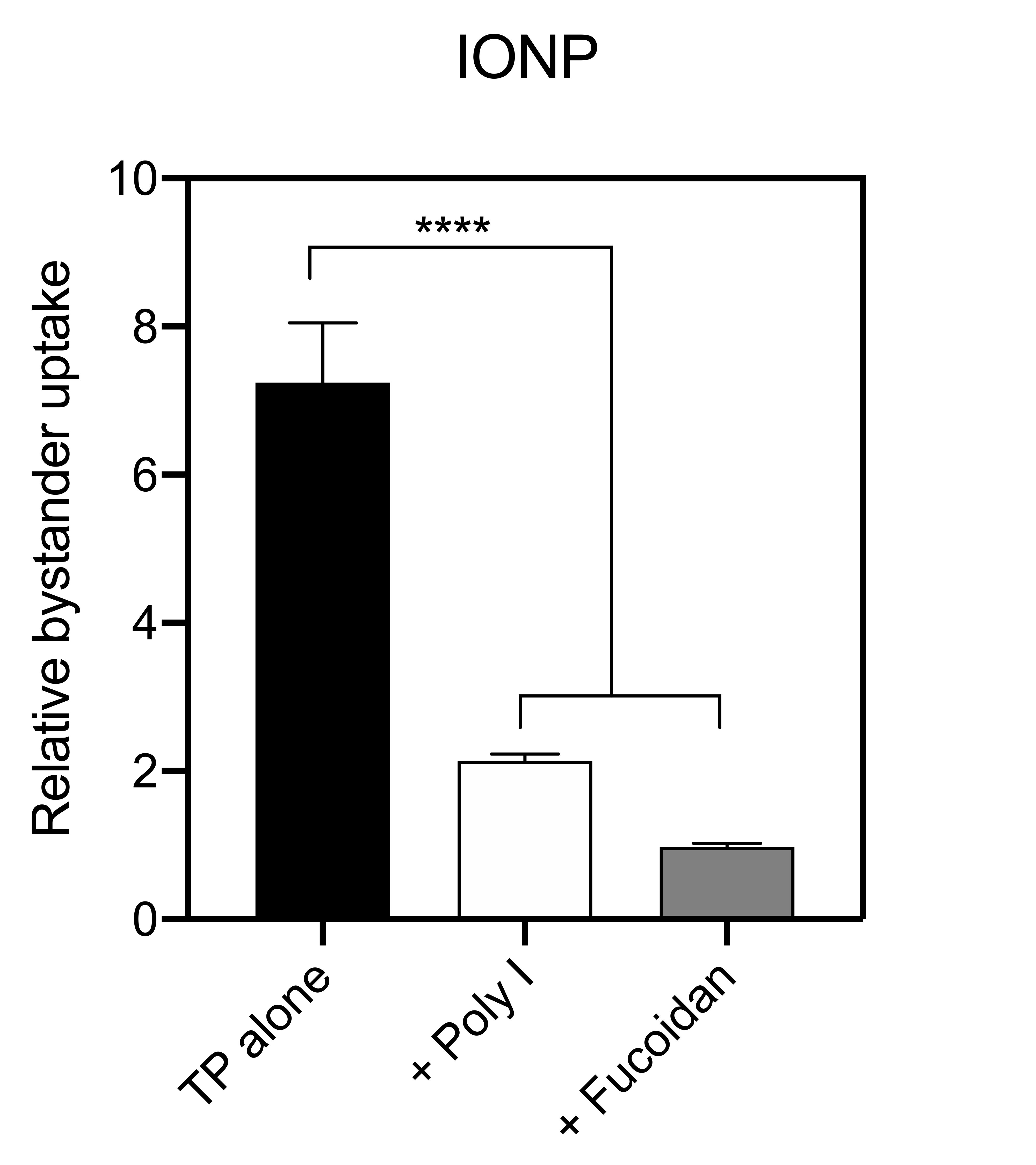


**Figure S3.** Dependence on scavenger receptors. (**A)** Blockage of TP uptake by two scavenger receptor agonists. Cells were incubated with 50 μg/mL poly I or 1500 μg/mL fucoidan together with TP peptide for 1 h, as described in the Methods. Cells were then washed, detached and subjected to flow cytometry. Fluorescence intensity of internalized TP peptide was normalized to that of cells alone (y-axis). Error bars, mean ± standard deviation (s.d.) (*n* = 3). One-way ANOVA with Tukey’s multiple comparisons test was performed. **** *P* < 0.0001 in comparison with the TP alone group. (**B)** Blockage of NP bystander uptake by two scavenger receptor agonists. As described in the Methods, cells were incubated with indicated bystander NPs (AgNPs, AuNPs and IONPs) + TP peptide alone, or together with poly I or fucoidan. Fluorescence intensity of bystander NPs per sample was quantified by flow cytometry and normalized to that of cells with bystander NPs alone (y-axis). Data were presented as mean ± s.d. (*n* = 3) and analyzed using one-way ANOVA with Tukey’s multiple comparisons test. ** *P* < 0.01, *** *P* < 0.001, **** *P* < 0.0001 in comparison with the TP alone group.

**A**


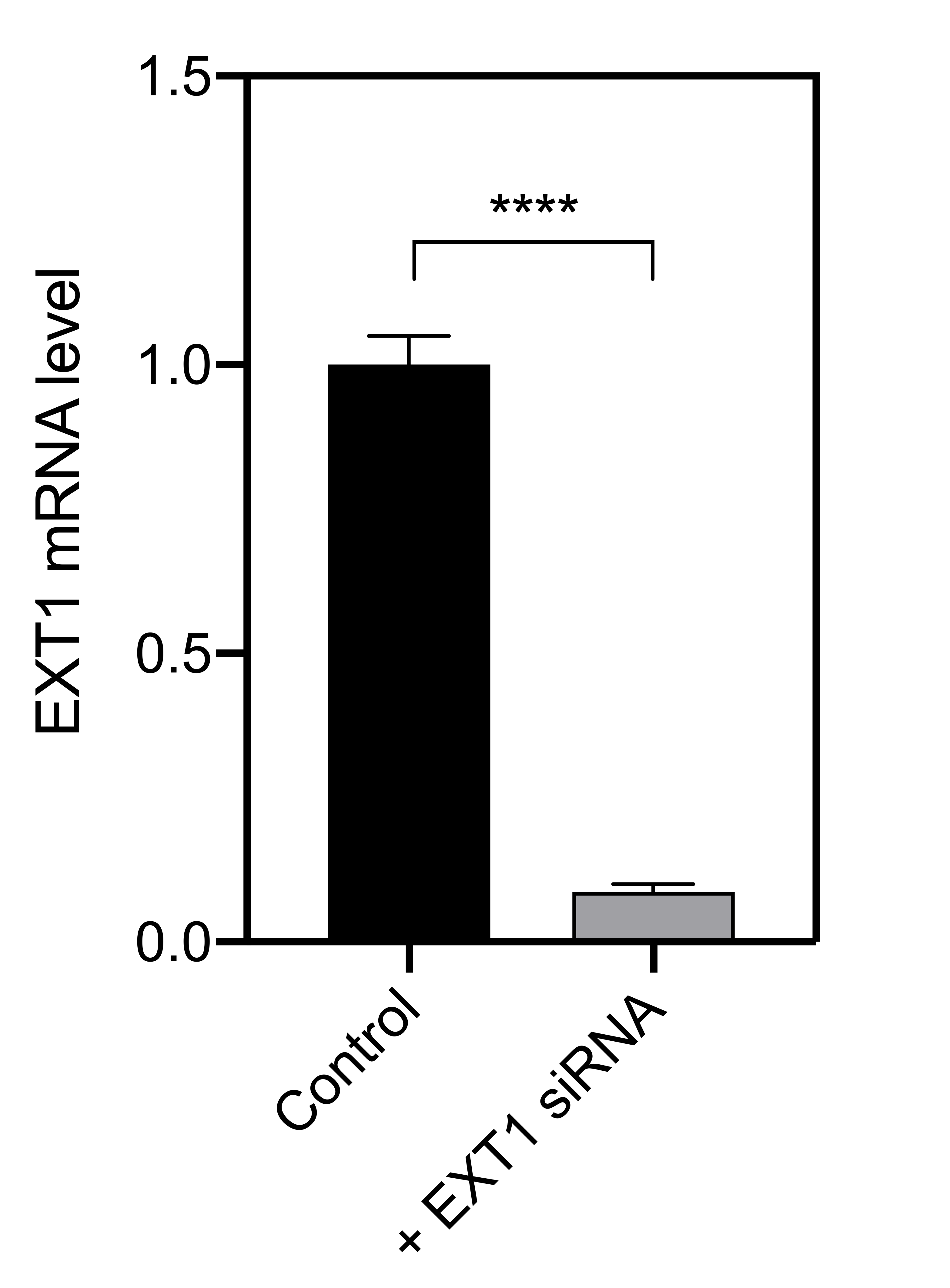


**B**


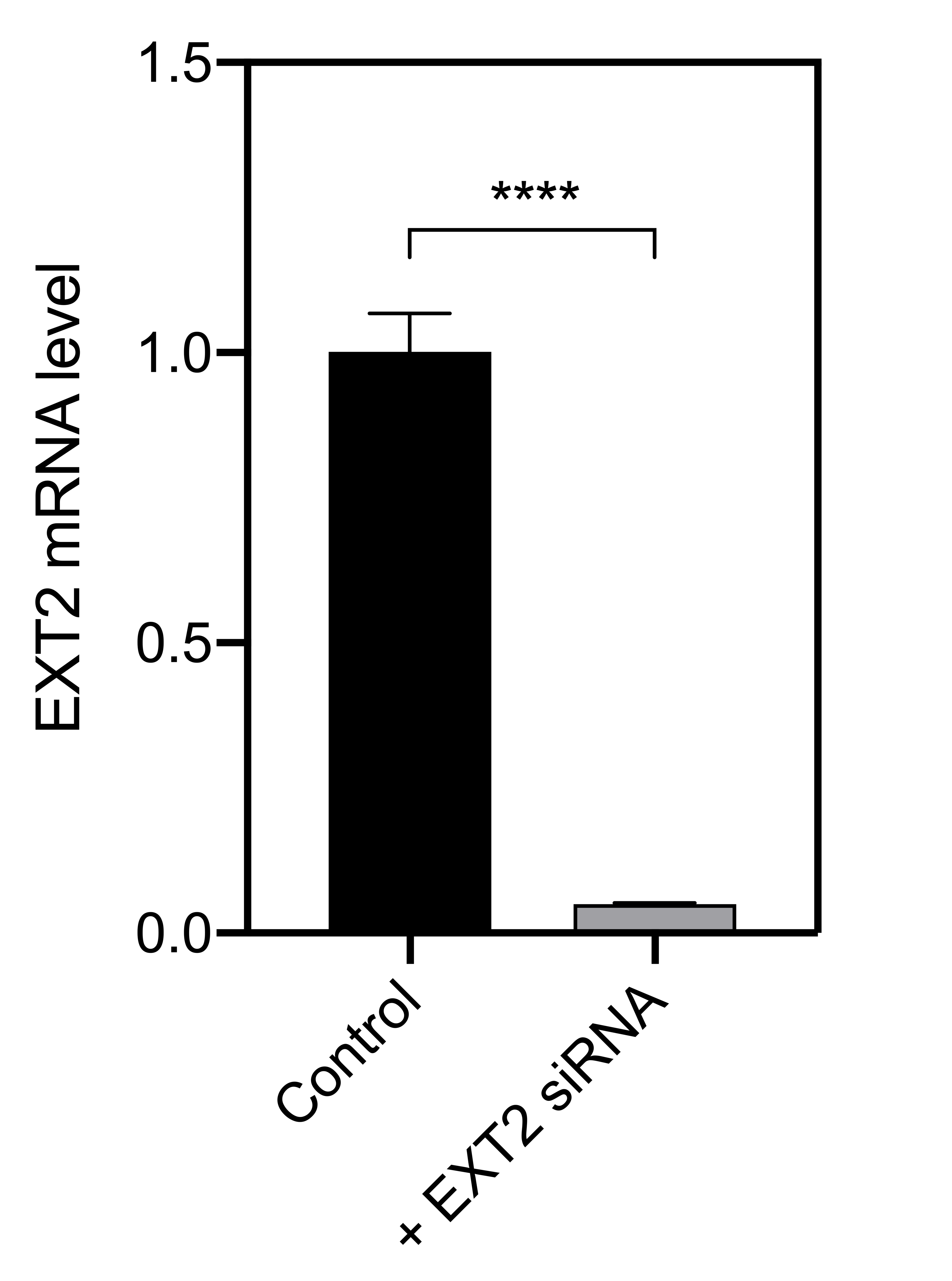


**C**


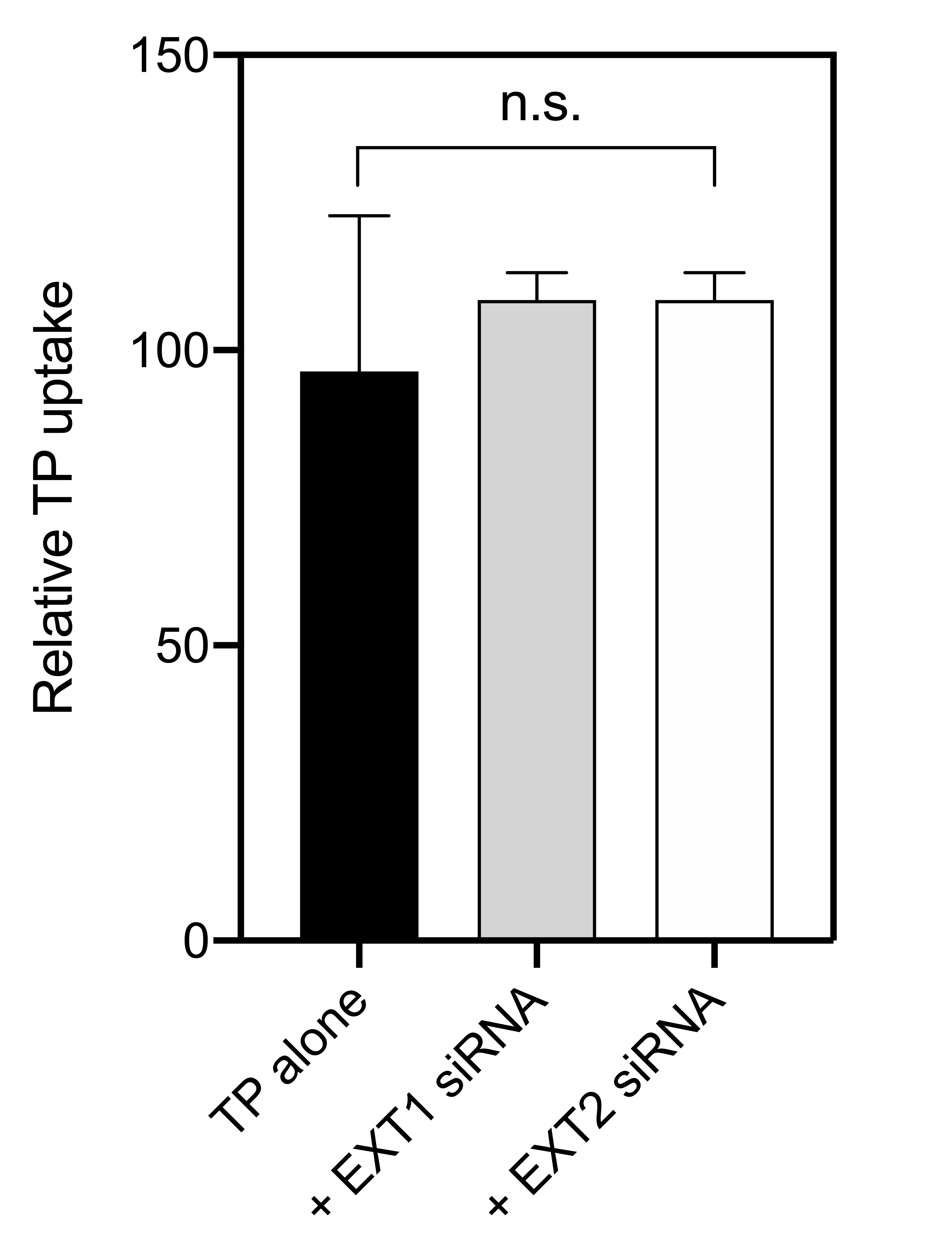

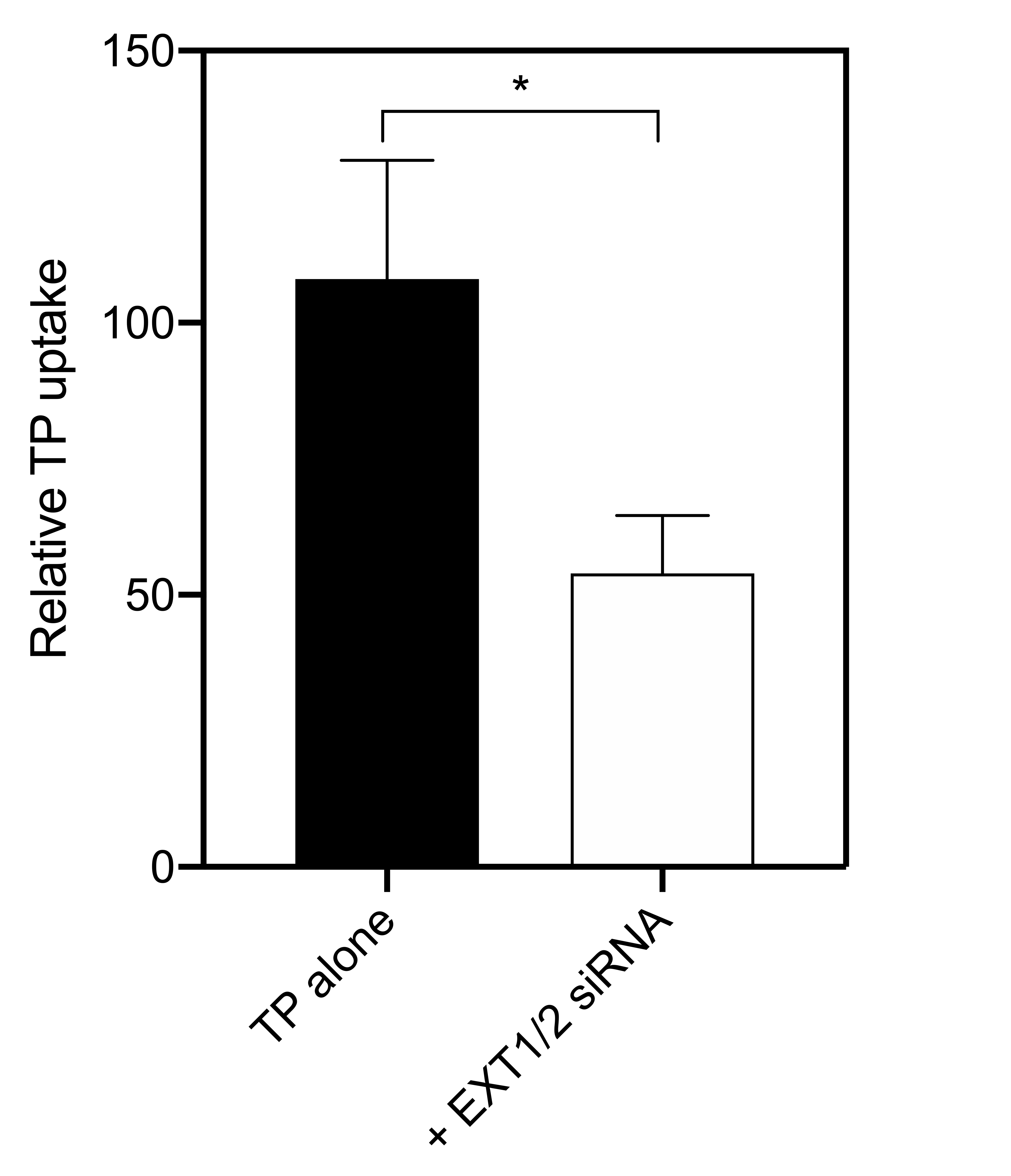

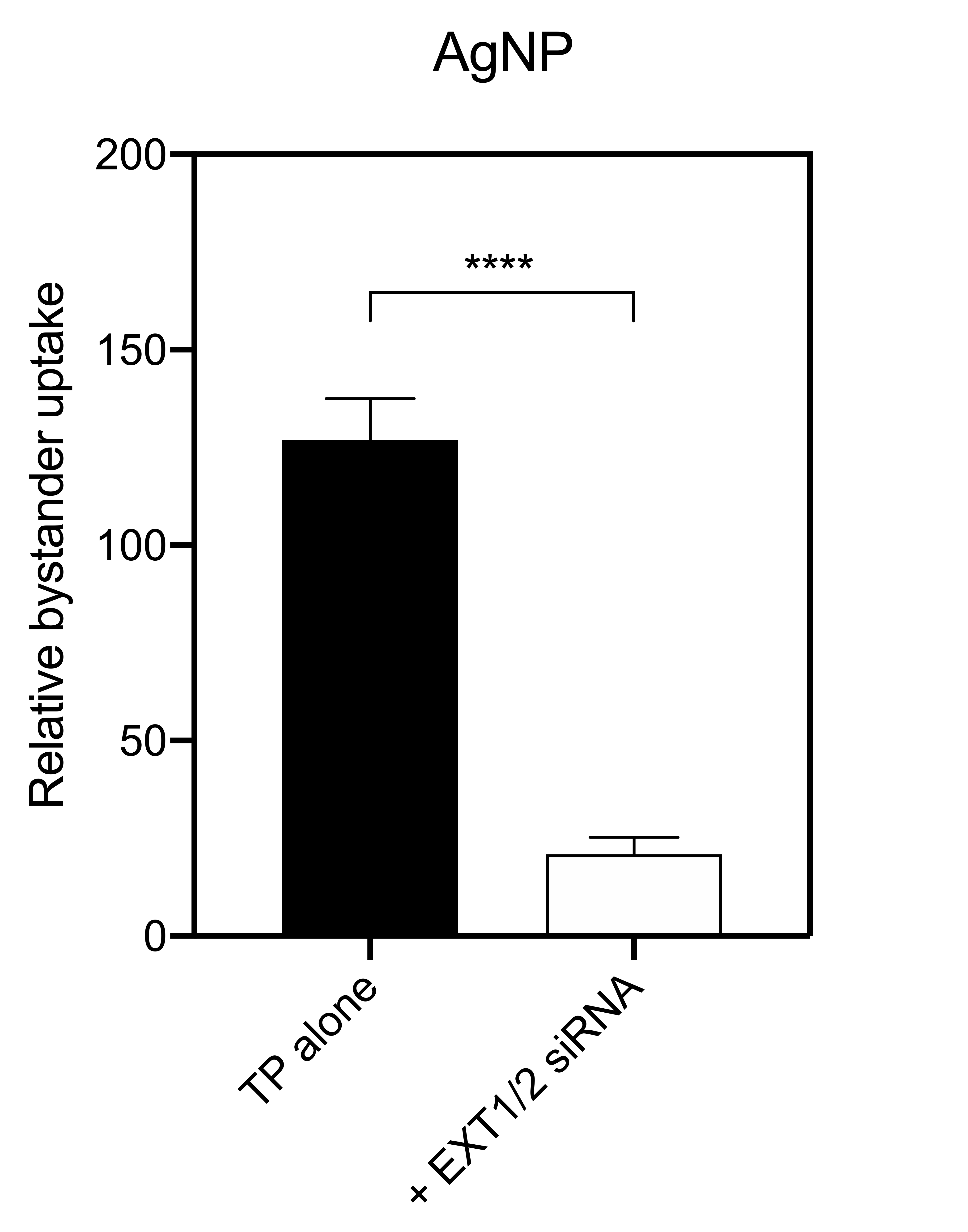


**D**

**Figure S4.** HSPG dependence. (**A)** The effect of EXT genes knockdown on RNA expression. Hela cells were transfected with EXT1 siRNA or EXT2 siRNA respectively for 48 h and mRNA expression levels of EXT1 and EXT2 were then analyzed by RT-PCR, as described in the Methods. Error bars, mean ± standard deviation (s.d.) (*n* = 3). Two tailed Student’s t-test was performed. **** *P* < 0.0001. (**B)** The effect of individual EXT gene knockdown on TP uptake. Hela cells were transfected with EXT1 siRNA or EXT2 siRNA respectively for 48 h and then incubated with 10 μM TP peptide for 1 h. Cells were washed, detached and subjected to flow cytometry. Fluorescence intensity of internalized TP peptide was normalized to that of cells alone (y-axis). Error bars, mean ± standard deviation (s.d.) (*n* = 3). One-way ANOVA with Tukey’s multiple comparisons test was performed. n.s., not significant. (**C)** Blockage of TP uptake by EXT1/2 genes knockdown. Hela cells were transfected with EXT1 siRNA together with EXT2 siRNA for 48 h, as described in the Methods. Flow cytometry was used to quantify the fluorescence intensity of internalized TP peptide which was further normalized to that of cells alone (y-axis). Error bars, mean ± standard deviation (s.d.) (*n* = 3). Two tailed Student’s t-test was performed., * *P* < 0.05. (**D)** Blockage of NP bystander uptake by EXT1/2 genes knockdown. Hela cells were transfected with both EXT1 and EXT2 siRNA for 48 h and then incubated with indicated bystander cargo (AgNP) + TP peptide for 1 h. Fluorescence intensity of bystander NPs per sample was quantified by flow cytometry and normalized to that of cells with bystander NPs alone (y-axis). Error bars, mean ± standard deviation (s.d.) (*n* = 3). Two tailed Student’s t-test was performed. **** *P* < 0.0001 in comparison with the TP alone group.

**Table 1.** List of scavenger receptors expressed in Hela cells.

| Gene | Gene description | NX |
| --- | --- | --- |
| *LRP1* | LDL receptor related protein 1 | 2.7 |
| *SCARB1* | Scavenger receptor class B member 1 | 22.4 |
| *MSR1* | Macrophage scavenger receptor 1 | 1.8 |
| *ACKR3* | Atypical chemokine receptor 3 | 23.4 |
| *SCARB2* | Scavenger receptor class B member 2 | 4.2 |
| *SCARA3* | Scavenger receptor class A member 3 | 55.7 |
| *TMPRSS3* | Transmembrane serine protease 3 | 7.9 |
| *PRSS12* | Serine protease 12 | 1.1 |
| *CD68* | CD68 molecule | 1.8 |
| *LOXL4* | Lysyl oxidase like 4 | 1.0 |
| *GBA* | Glucosylceramidase beta | 11.0 |
| *LGALS3BP* | Galectin 3 binding protein | 23.1 |
| *LOXL2* | Lysyl oxidase like 2 | 2.3 |
| *PGBD1* | PiggyBac transposable element derived 1 | 5.8 |
| *TINAGL1* | Tubulointerstitial nephritis antigen like 1 | 1.4 |
| *ZNF444* | Zinc finger protein 444 | 6.1 |
| *OLR1* | Oxidized low density lipoprotein receptor 1 | 1.6 |
| Scavenger receptor genes are considered as specifically expressed in Hela cells if NX >= 1.0. NX, the consensus normalized expression value. The database used for this work is The Human Protein Atlas. | | |
